# Multi-Ray Modeling of Ultrasonic Sensors and Application for Micro-UAV Localization in Indoor Environments

**DOI:** 10.3390/s19081770

**Published:** 2019-04-13

**Authors:** Lingyu Yang, Xiaoke Feng, Jing Zhang, Xiangqian Shu

**Affiliations:** School of Automation Science and Electrical Engineering, Beihang University, Beijing 100191, China; yanglingyu@buaa.edu.cn (L.Y.); 18580530093@163.com (X.F.); shuxiangqian@163.com (X.S.)

**Keywords:** indoor location, multi-ray model of ultrasonic sensors, micro-UAV, extended Kalman filter

## Abstract

Due to its payload, size and computational limits, localizing a micro air vehicle (MAV) using only its onboard sensors in an indoor environment is a challenging problem in practice. This paper introduces an indoor localization approach that relies on only the inertial measurement unit (IMU) and four ultrasonic sensors. Specifically, a novel multi-ray ultrasonic sensor model is proposed to provide a rapid and accurate approximation of the complex beam pattern of the ultrasonic sensors. A fast algorithm for calculating the Jacobian matrix of the measurement function is presented, and then an extended Kalman filter (EKF) is used to fuse the information from the ultrasonic sensors and the IMU. A test based on a MaxSonar MB1222 sensor demonstrates the accuracy of the model, and a simulation and experiment based on the ThalesII MAV platform are conducted. The results indicate good localization performance and robustness against measurement noises.

## 1. Introduction

Micro air vehicles (MAVs) are a type of drone and are approximately the size of a person’s hand. This property makes them easy to pack and allows them to be flown indoors. One of the fundamental problems of autonomous indoor flight is the localization ability. This problem has become more severe due to the strict restrictions on the size and weight of MAVs. Thus, how to utilize low-cost and lightweight sensor resources to locate MAVs in complex and ever-changing indoor environments is a hot and challenging issue.

Many indoor localization technologies have been developed to achieve indoor localization, such as localization based on ranging sensors [1,2,3], Bluetooth [4], inertial measurement units (IMUs), cameras, ultra wide band (UWB) [5], wireless local area network (WLAN) [6], ZigBee [7] and radio frequency sensors [8]. In this paper, the above approaches can be divided into two types according to whether the main localization sensors are placed on the unmanned aerial vehicle (UAV): onboard-sensor-based approaches and offboard-sensor-based approaches. The offboard-sensor-based approaches, such as Cricket developed by MIT, require some equipment, such as the beacons or motion capture cameras, to be prearranged in the UAV’s flight environment; thus, such approaches have good positioning accuracy in known environments.

The onboard-sensor-based approaches, which do not require the assistance of external devices, can be applied to unknown environments. In [9], the data from the IMUs and lidar are used as inputs to the odometer, and the position of the UAV and the map are given simultaneously. In [10], a landmark-based method is introduced. In this method, some simply shaped objects, such as walls, corners and edges, are chosen as landmarks. Additionally, 16 ultrasonic sensors are mounted around the mobile robot to identify and measure the distance to the landmarks. Then, the robot’s position can be obtained when two geometrical elements are successfully identified. In [11], extracted and matched scale invariant feature transform (SIFT) features are used to construct nonlinear least squares problems, and then the pose of the UAV is solved by the Gauss-Newton method, using an IMU to estimate the initial value of the solution. In [12], the Harris corner detection algorithm is used to detect the corner points, and the corner points of two adjacent images are matched to obtain an optimized objective function; then, the LM algorithm is used for nonlinear optimization, and finally, the pose of the UAV is obtained. In [13], a lamp on the ceiling is used as a landmark, and through the extraction of feature points on the lamp, real-time localization can be realized by combining the relevant information of the landmark in the database. In [14], lidar data are segmented using KD trees, and then the PLICP algorithm is used to match the point sets of two adjacent scans; the error equation is constructed according to the distance between these matching points. Through the iterative solution of the equation, the rotation and translation of two adjacent scans are calculated, and then the position of the robot is estimated. In [15], the author uses a planar object for positioning. First, the laser data are segmented and subject to plane fitting. Then, a variant of the hill-climbing algorithm is used to match the planes in data of two adjacent scans. Finally, three successful matching planes are selected to calculate the location of the robot based on the geometric relationship.

Considering their limited size and load, very few approaches are available for MAVs. The lidar-based and depth-camera-based approaches are too large or too heavy. Although a monocular camera or binocular camera can be small and light enough for a MAV, the corresponding image processing device is also unacceptable for being installed in a MAV, at least at present. Compared with the above approaches, ultrasonic range sensors have advantages in terms of size and weight, making them one of the best choices for the localization task.

In [16], a few well-known ultrasonic localization systems, including Cricket, BUZZ and Dolphin, are investigated with a comparison of the systems in terms of performance, accuracy and limitations. The accuracies of the above systems range from 1.5 cm to 10 cm; however, these positioning approaches require special application conditions, such as arranging transmitters in the environment, time synchronization processing, and powerful computing capabilities. Thus, they are hard to apply in MAVs. In [3], a ultrasonic-beacon-based approach is proposed to replace the role of GPS, it consists several stationary beacons and a mobile beacon and has a good balance between the weight and accuracy. However, it still needs the assistance of external devices, i.e, the stationary beacons, which may limits it application. Ref. [2] discusses a possible way to map an unknown indoor environment by using 3 ultrasound modules. Ref. [17] summarizes several commonly used sonar models, such as the centerline model, the occupancy grids model, the polygon model and the arc model. In [18], an improved wedge model of the sonar sensor model is given, and a probabilistic measurement model that takes the sonar uncertainties into account is defined according to the experimental characterization. Experiments are conducted based on a Pioneer 3-DX robot equipped with 16 Polaroid ultrasonic range finders. However, a certain number of sensors are required to obtain satisfactory positioning accuracy, which is hard to apply to a light MAV.

In this paper, a novel beaconless localization approach is proposed and a multi-ray ultrasonic sensor model is presented to provide a rapid and accurate approximation of the complex beam pattern of ultrasonic sensors. Additionally, four ultrasonic sensors are used to achieve position estimation. The proposed localization approach is suitable for MAVs in terms of weight and computation.

This paper is organized as follows. The ThalesII MAV platform is presented in Section 2. The multi-ray model of ultrasonic sensors is given in Section 3. The MAV system is modeled in Section 4. Section 5 presents the localization algorithm based on EKF. In the last section, simulation and experimental results are presented to validate the proposed algorithm.

## 2. The Micro-UAV Platform

**Figure 1:**
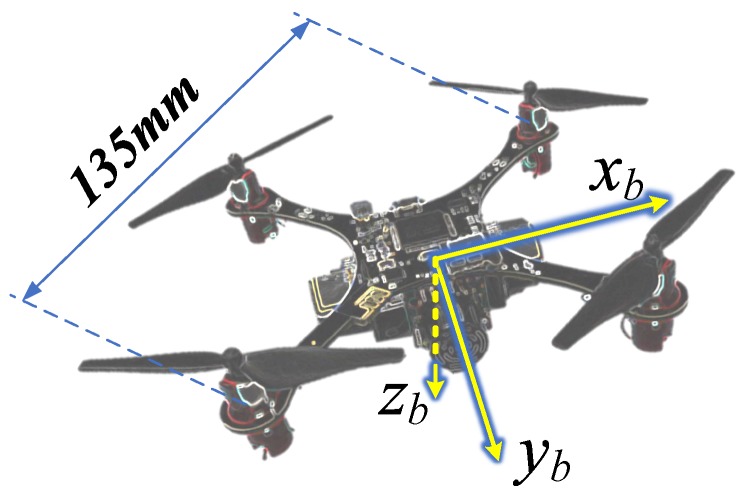
The ThalesII micro air vehicle (MAV) platform with body frame axis orientation.

The ThalesII indoor MAV platform, shown in Figure 1, is the second generation of the Thales series created by the our group [19]. The MAV has the advantages of small size and light weight, and it can fly for about 4 min with a 400 mA battery.The weight of the ThalesII platform is approximately 75 g, which consists of the airframe (15 g), the battery (12 g), 4 motors and propellers (24 g) and 4 MB1222 sonar range finders (24 g), and its diagonal length is 135 mm (motor to motor).

**Figure 2:**
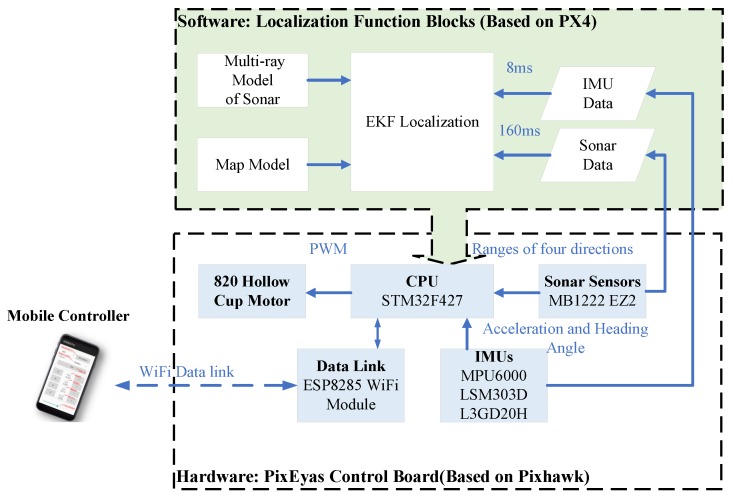
The system architecture of ThalesII MAV platform.

The system architecture of the ThalesII MAV platform is shown in Figure 2, the lower part of the architecture shows the main hardware components, and it is a modified version based on the open source hardware Pixhawk [20]. The powerful ARM STM32F427 is used to perform the calculation and the ESP8285 WiFi module is used to communicate with the mobile controller. Four 820-hollow-cup-motors are used to drive the 55 mm propellers. The angular velocity and movement acceleration are measured by an MPU6000 IMU sensor, and the heading angle is provided by an LSM303 magnetic sensor; both sensors have a sampling period of 8 ms.

**Figure 3:**
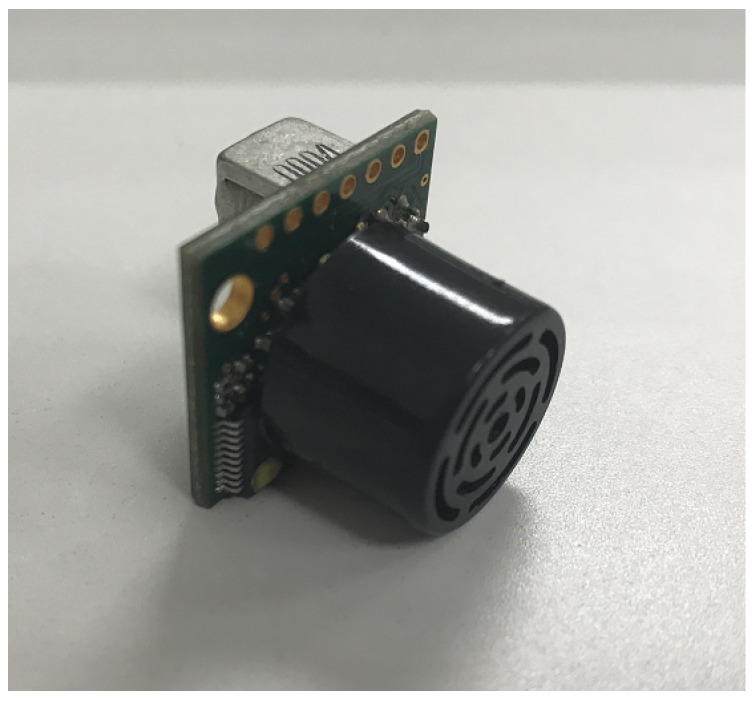
The MB1222 I2CXL-MaxSonar-EZ2 sonar range finder.

Considering the size and load limitations, some widely used precise distance measurement approaches, such as the laser range finder and the depth camera, cannot be applied in the MAV platform. In the ThalesII platform, four MB1222 I2CXL-MaxSonar-EZ2 range finders are installed on the bottom of the MAV. They are installed perpendicular to each other, as shown in Figure 3. Thus, the ranges of four directions can be provided in a single measurement.

The features of the MB1222 I2CXL-MaxSonar-EZ2 range finder include centimeter resolution, an excellent compromise between sensitivity and side object rejection, short to long distance detection, range information from 20 cm to 765 cm, up to a 40 Hz read rate, and an I2C interface [21]. Thus, this sensor is one of the best choices for the localization task. The other features of the MAV platform are shown in Table 1.

The operating system running on the flight control board is the open source software PX4. It is easy to develop customized tasks, and all the data during the flight period are easy to store. The main functions of the proposed localization algorithm are shown as the upper part in Figure 2.

## 3. Modeling of the Ultrasonic Sensors

Ultrasonic sensors are based on the time of flight to measure distance and return a range. However, this range is not the straight line distance to an obstacle; rather, it is the distance to the point that has the strongest reflection. This point could be anywhere along the perimeter of the sensor’s beam pattern [17,22], which makes the modeling of ultrasonic sensors a complex issue, particularly for online computing.

**Figure 4:**
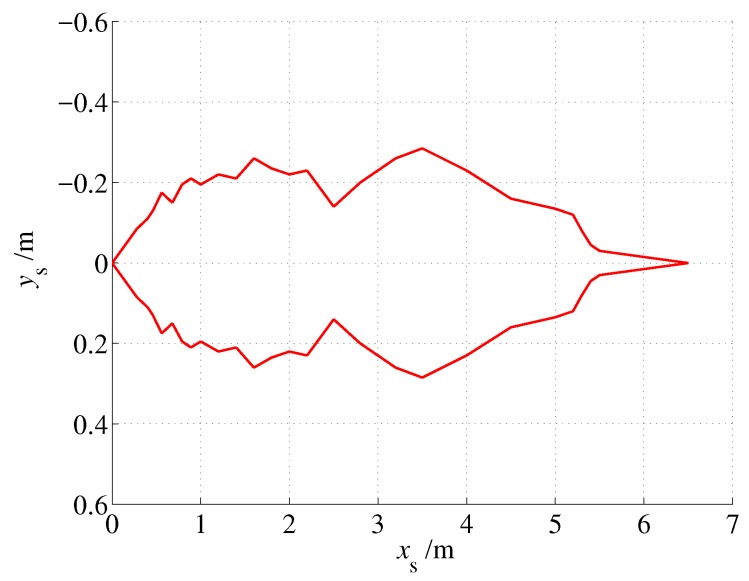
The beam pattern of MaxSonar MB1222 sonar sensor.

Figure 4 shows the detection area of the MaxSonar MB1222 sonar sensor; it is obtained by placing and measuring a plastic plate at predefined grid points in front of the ultrasonic sensor.

**Figure 5:**
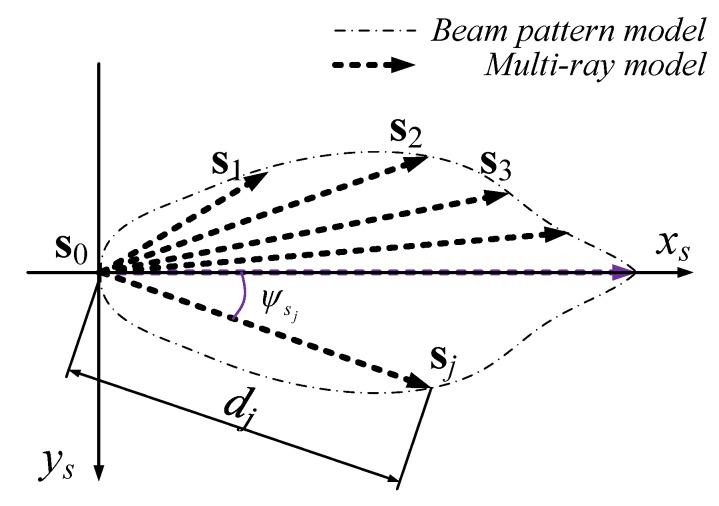
The sonar coordinate system and the multi-ray model of sonar.

As shown in Figure 4, the 2D beam pattern of the MB1222 sensor was approximated as an irregular polygon. To reduce the computational load of the polygon model, a multi-ray model is proposed, and the beam pattern is approximated by a ray group that starts from the origin, as shown in Figure 5.

Then, the ultrasonic 2D multi-ray model S can be formulated as a ray group as

(1) $$\left\{\begin{array}{c} S=\{S_{1},S_{2},\ldots ,S_{k}\}\\ S_{1}=\bar{s_{0}s_{1}},S_{2}=\bar{s_{0}s_{2}},\cdots ,S_{k}=\bar{s_{0}s_{k}} \end{array}\right.,$$

where $s_{0}$ represents the sonar sensor’s position and $s_{j}$ is the end point of the *j*-th ray. Thus, for a known obstacle O, the model output *l* is obtained through a two-step calculation. First, a set of all the intersections of O and S is calculated as

(2) $$R=\{r_{1},r_{2},\ldots ,r_{q}\}=S⨂O,$$

and then *l* is given as

(3) $$l=\left\{\begin{array}{cc} \min_{r_{i}}\{∥r_{i}-s_{0}{∥}_{2}\} & R\neq \emptyset \\ l_{\max} & R=\emptyset \end{array}\right..$$

Equation (3) follows the principle that the ultrasonic sensor provides the nearest measurement of all detections, and a predefined value $l_{\max}$ is given if there is no intersection between S and R.

**Figure 6:**
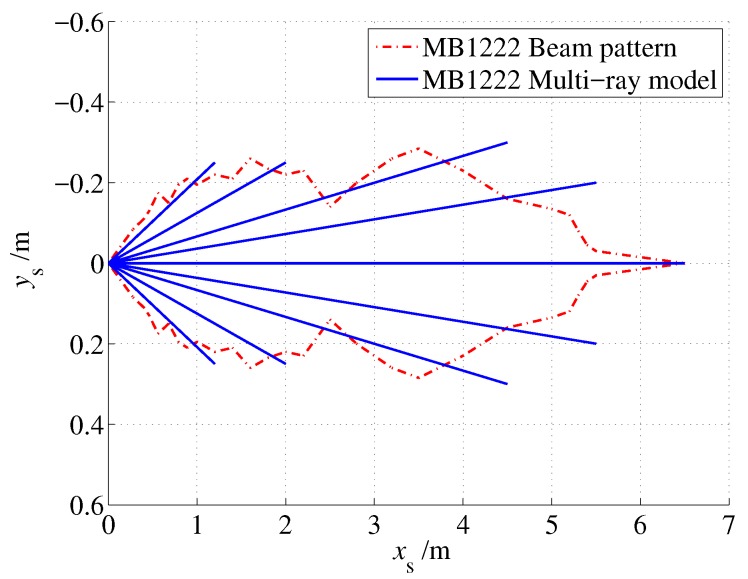
The multi-ray model of the MaxSonar MB1222 sonar sensor.

Based on the beam pattern of the MaxSonar MB1222 sonar sensor, the multi-ray model was given as shown in Figure 6. Nine rays were used to approximate the detection zone of MB1222. Note that the far ends of the rays were selected slightly beyond the edge to obtain better coverage of the detection zone.

**Figure 7:**
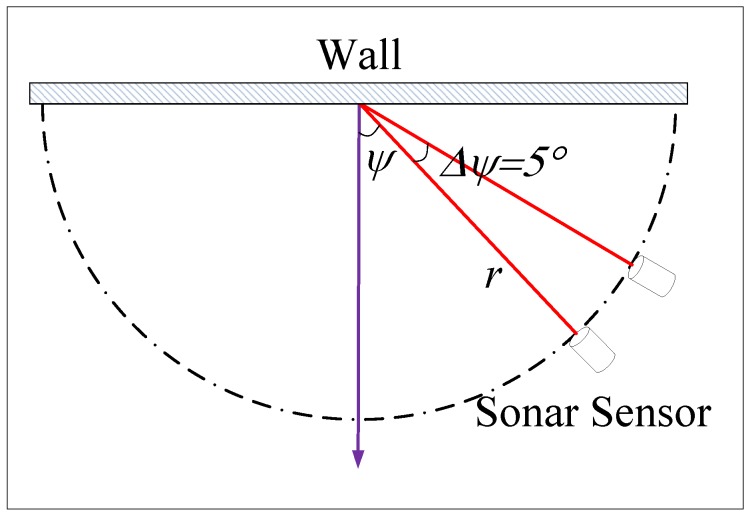
Test scheme.

To test the fitness of the multi-ray model and the actual sensor measurement, a comparative test was performed between the proposed model and the MB1222 sensor, as shown in Figure 7. The sensor was placed on the edge of a semicircle with radius *r*, pointing to the center of the circle, and the angle ψ was then increased in five-degree steps. The actual measurement $l_{t}$ is shown in Table 2. The corresponding output of the multi-ray model $l_{m}$ is presented in Table 3. The modeling error $l_{e}$ is presented in Table 4.

As shown in Table 2,

(1) The measurement had a constant offset of approximately 3 cm to 4 cm, even in ψ=0, i.e., the sensor is perpendicular to the wall.

(2) The maximum detection angles varied with the distances to the wall. The farther the sensor was from the wall, the narrower the detection angle. The half-side detection angle was close to 0 when the distance exceeded 5.9 m, and it reached approximately 35 degrees when *r* was less than 1.2 m.

For comparison, the 3 cm offset was subtracted from the output of the model, and the model error was defined as $l_{e}=l_{t}-l_{r}-3$ cm, as shown in Table 3 and Table 4. As shown, in most cases, the model error was less than 1 cm, and the maximum model error was 2 cm. Considering that the minimum resolution of the sensor was 1 cm, the proposed model had good fitness with the actual sensor for indoor localization.

Note that obvious angular constraint characteristics were observed in the measurements of ultrasonic sensors; however, we did not introduce the angular constraint in the proposed model, which was a consideration for reducing the calculation load. Because the constraint involves calculating the angles between all line segments of S and O, it may lead to a significant increase in the calculation load. In an alternative approach, the jump filter, was used to solve this problem, which will be presented in Section 5.

## 4. Modeling of the MAV System

**Figure 8:**
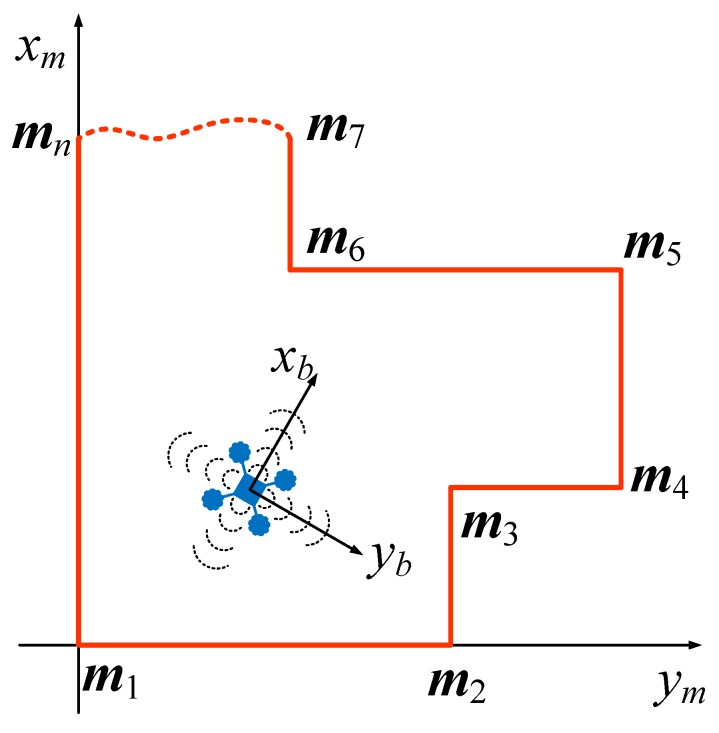
The map and body coordinate systems.

To describe the motion of the MAV, the map coordinate system $O_{m-x_{m},y_{m},z_{m}}$ and the body coordinate system $O_{b-x_{b},y_{b},z_{b}}$ were introduced. The map coordinate system $O_{m-x_{m},y_{m},z_{m}}$ was fixed to the earth, and its origin is located at the starting corner $m_{1}$ of the map M. The body coordinate system $O_{b-x_{b},y_{b},z_{b}}$ was fixed to the MAV (in strapdown configuration), as shown in Figure 8.

The 2D polygonal map M can be formed as a set of line segments as

(4) $$\left\{\begin{array}{cc} M & =\{M_{1},M_{2},\ldots ,M_{n}\}\\ M_{1} & =\bar{m_{1}m_{2}},M_{2}=\bar{m_{2}m_{3}},\ldots ,M_{n}=\bar{m_{n}m_{1}} \end{array}\right.,$$

where $\bar{ab}$ represents a line segment connecting points a and b. $m_{i}={\left[m_{i_{x}},m_{i_{y}}\right]}^{⊤},\left(i=1,\ldots ,n\right)$ is the position of the $i^{th}$ corner in the map coordinate system.

The direct cosine matrix (DCM) is used to translate the acceleration from the body frame to the map frame.

(5) $$R_{bw}=\left[\begin{array}{ccc} \cos\theta \cos\psi & \sin\phi \sin\theta \cos\psi -\cos\phi \sin\psi & \cos\phi \sin\theta \cos\psi +\sin\phi \sin\psi \\ \cos\theta \sin\psi & \sin\phi \sin\theta \sin\psi +\cos\phi \cos\psi & \cos\phi \sin\theta \sin\psi -\sin\phi \sin\psi \\ -\sin\theta & \cos\theta \sin\phi & \cos\theta \cos\phi \end{array}\right],$$

where ${\left[\phi ,\theta ,\psi \right]}^{⊤}$ are the roll, pitch and yaw angles, respectively.

Then, the accelerations on the body frame can be transferred to the map frame by

(6) $$a_{w}=R_{bw}a_{b}+G,$$

where $G={\left[0,0,g\right]}^{⊤}$ is the gravity vector in the map frame. Therefore, the discrete-time state-space model of the MAV is given by

(7) $$x\left(k+1\right)=Ax\left(k\right)+Ba_{w}\left(k\right)$$

(8) $$x\left(k\right)=\left[\begin{array}{c} p_{x}\\ v_{x}\\ p_{y}\\ v_{y} \end{array}\right],A=\left[\begin{array}{cccc} 1 & t_{\mathrm{imu}} & 0 & 0\\ 0 & 1 & 0 & 0\\ 0 & 0 & 1 & t_{\mathrm{imu}}\\ 0 & 0 & 0 & 1 \end{array}\right],B=\left[\begin{array}{cc} 0.5t_{\mathrm{imu}}^{2} & 0\\ t_{\mathrm{imu}} & 0\\ 0 & 0.5t_{\mathrm{imu}}^{2}\\ 0 & t_{\mathrm{imu}} \end{array}\right],$$

where $t_{\mathrm{imu}}$ represents the sampling period of the IMU and $v\left(k\right)={\left[v_{x}\left(k\right),v_{y}\left(k\right)\right]}^{⊤}$ and $p\left(k\right)={\left[p_{x}\left(k\right),p_{y}\left(k\right)\right]}^{⊤}$ are the velocity vector and position vector in the map frame at step *k*, respectively. The output of the MAV system was the measurement of multiple sonar sensors, which is defined as

(9) l(k)=h(x(k),ψ(k),S,M),

where $l={\left[l_{1},l_{2},l_{3},l_{4}\right]}^{⊤}$ is the measurement vector of sonar sensors, and h() is a nonlinear function of p(k), ψ(k), the sonar model S and the map of the working area M. To obtain the measurements of the sonar sensors, one needs to represent the sonar’s model S in the map coordinate system. Since S is a set of line segments, this transformation can be achieved by representing the endpoints of line segments as

(10) $$\left\{\begin{array}{c} s_{0}=p+d_{0}{\left[\cos\left(\psi +\psi_{s_{0}}\right),\sin\left(\psi +\psi_{s_{0}}\right)\right]}^{⊤}\\ s_{j}=s_{0}+{\left[d_{j}\cos\left(\psi +\psi_{s_{0}}+\psi_{s_{j}}\right),d_{j}\sin\left(\psi +\psi_{s_{0}}+\psi_{s_{j}}\right)\right]}^{⊤}\left(j=1\ldots k\right), \end{array}\right.$$

where p and ψ denote the UAV’s position and heading angle in the map coordinate system, respectively. $\psi_{s_{0}}$ is the heading angle of sonar in the body coordinate system, and $d_{0}$ is the length between the origins of the body frame and of the sonar frame. Additionally, $d_{j}$ and $\psi_{s_{j}}$ are the length and the angle of the *j*th ray in the sonar coordinate system, respectively. Then, the ultrasonic sensor’s measurement *l* is given by Equations (3) and (11).

(11) $$R=\{r_{1},r_{2},\ldots ,r_{q}\}=S⨂M,$$

In particular, among all the intersections, the one that minimizes Equation (3) is defined as the “active intersection” $r^{a}$, and terms “active ray” $s^{a}$ and “active wall” $M^{a}$ are introduced to represent the corresponding ray and the corresponding wall with the active intersection.

## 5. Indoor Localization Method Based on the EKF

As shown in Equation (3), the measurement function of the system is a nonlinear and discontinuous function; thus, using the EKF rather than the traditional Kalman filter is a feasible way to estimate the location of the MAV. The key issue is to solve the Jacobian matrix of Equation (3).

The gradient matrix of the function h with respect to x at step *k* is given by

(12) $$H\left(k\right)={\left.\frac{\partial l}{\partial x}\right|}_{x(k),\psi (k),S,M}.$$

**Figure 9:**
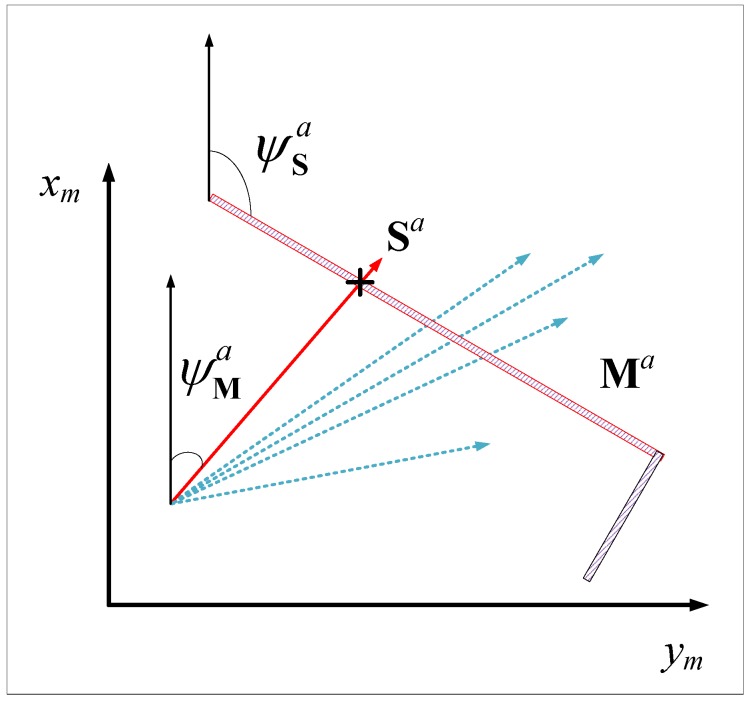
The active ray and active wall.

Based on the multi-ray model, the Jacobian matrix can be calculated by geometric methods. At time *k*, suppose that the relationship between the sonar model and the map is as shown in Figure 9. Additionally, assume that the active ray $S^{a}$ and the active ray $M^{a}$ remain unchanged. The Jacobian matrix can then be given as

(13) $$\left\{\begin{array}{cc} \frac{\partial l_{i}}{\partial v_{x}} & =0\\ \frac{\partial l_{i}}{\partial v_{y}} & =0\\ \frac{\partial l_{i}}{\partial p_{x}} & =\frac{-\sin\psi_{M_{i}}^{a}\left(k\right)}{\sin(\psi_{M_{i}}^{a}\left(k\right)-\psi_{S_{i}}^{a}\left(k\right))}\\ \frac{\partial l_{i}}{\partial p_{y}} & =\frac{\cos\psi_{M_{i}}^{a}\left(k\right)}{\sin(\psi_{M_{i}}^{a}\left(k\right)-\psi_{S_{i}}^{a}\left(k\right))} \end{array}\right.i=1,2,3,4,$$

where $\psi_{S_{i}}^{a}$ and $\psi_{M_{i}}^{a}$ represent the yaw angles of the “active ray” and the “active wall” of the $i^{th}$ ultrasonic sensor in the map frame. In addition, $\frac{\partial l_{i}}{\partial p_{x}}$ and $\frac{\partial l_{i}}{\partial p_{y}}$ were set to zeros if there was no obstacle in the detection range of the $i^{th}$ ultrasonic sensor. Then, the MAV’s position can be obtained through a standard EKF procedure as

(14) $$\left\{\begin{array}{cc} \hat{x}\left(k|k-1\right) & =A\cdot \hat{x}\left(k-1|k-1\right)+B\cdot a_{w}\\ P(k|k-1) & =A\cdot P\left(k-1|k-1\right)\cdot A^{⊤}+Q \end{array}\right..$$

(15) $$\left\{\begin{array}{cc} \hat{x}\left(k|k\right) & =\hat{x}\left(k|k-1\right)\\ P(k|k) & =P(k|k-1) \end{array}\right..$$

(16) $$\left\{\begin{array}{cc} \hat{l}\left(k\right) & =h(\hat{x}\left(k|k-1\right),\psi \left(k\right),S,M)\\ K(k) & =P\left(k|k-1\right)H{\left(k\right)}^{⊤}{\left[H\left(k\right)P\left(k|k-1\right)H{\left(k\right)}^{⊤}+R\right]}^{-1}\\ \hat{x}\left(k|k\right) & =\hat{x}\left(k|k-1\right)+K\left(k\right)\left[l\left(k\right)-\hat{l}\left(k\right)\right]\\ P(k|k) & =[I-K(k)H(k)]P(k|k-1) \end{array}\right..$$

Note that Equation (3) is a piecewise continuous function, and its output may jump in some conditions, such as if $S^{a}$ changes, $M^{a}$ changes or $S^{a}$ and $M^{a}$ change simultaneously. In addition, as mentioned in Section 3, if the angle between $S^{a}$ and $M^{a}$ exceeds the detection angle constraint, it may also lead to a significant deviation between l(k) and $\hat{l}\left(k\right)$. Similar results can also occur when the sensor occasionally malfunctions. Considering that the above cases will lead to a significant change in the term $l\left(k\right)-\hat{l}\left(k\right)$, a jump filter is given to solve this problem as

(17) $$\left\{\begin{array}{cc} K_{f}\left(k\right) & =K\left(k\right)\cdot diag({\left[\lambda_{1}\left(k\right),\lambda_{2}\left(k\right),\lambda_{3}\left(k\right),\lambda_{4}\left(k\right)\right]}^{⊤})\\ \lambda_{i}\left(k\right) & =\left\{\begin{array}{cc} 1 & |l_{i}\left(k\right)-{\hat{l}}_{i}\left(k\right)|\leq \epsilon \\ 0 & |l_{i}\left(k\right)-{\hat{l}}_{i}\left(k\right)|>\epsilon \end{array}\right.,i=1\ldots 4 \end{array}\right.,$$

where ϵ is a predesigned threshold. Therefore, if the measurement $l_{i}\left(k\right)$ is significantly different from its prediction ${\hat{l}}_{i}\left(k\right)$, i.e., $|l_{i}\left(k\right)-{\hat{l}}_{i}\left(k\right)|\leq \epsilon$, the corresponding measurement will be filtered out from the estimation.

**Figure 10:**
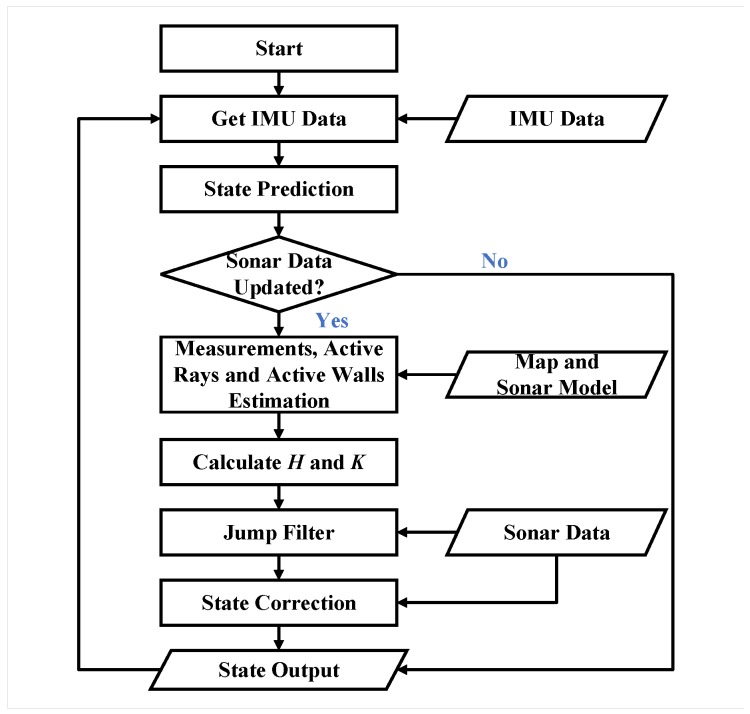
The extended Kalman filter (EKF) flowchart.

The flow chart of the indoor localization algorithm is shown in Figure 10.

## 6. Experiment

We thoroughly evaluate the proposed positioning algorithm using both a simulation and actual implementation.

### 6.1. Simulation Result

**Figure 11:**
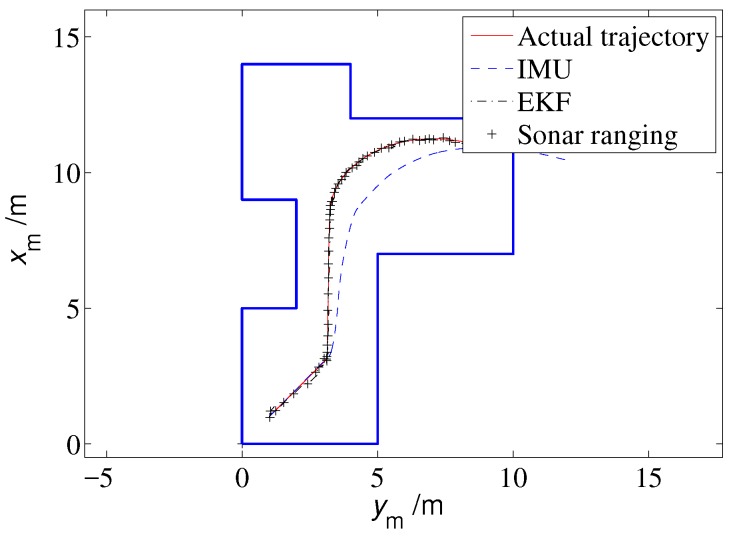
Localization results (simulation).

The localization algorithm developed in this paper was first tested through a simulation. To perform the simulation, a polygon a priori map is given as shown in Figure 11, and the sampled data of the accelerometer and the magnetic heading sensor are formed as

(18) $$\begin{array}{c} a_{b}={\bar{a}}_{b}+N\left(0,V_{a}\right),\\ \psi =\bar{\psi }+N\left(0,V_{\psi }\right), \end{array}$$

where ${\bar{a}}_{b}$ and $\bar{\psi }$ are the true acceleration and the true heading angle of the MAV, and $N(0,V_{a})$ and $N(0,V_{\psi })$ are the corresponding Gaussian noises with variances of $V_{a}$ and $V_{\psi }$.

For a MAV in this map, since the position, the heading angle, the map and the ultrasonic model are known, the ultrasonic theoretical measurement $\bar{l}$ is known. We also add a Gaussian noise with variance $V_{l}$ to it as

(19) $$l=\bar{l}+N\left(0,V_{l}\right),$$

**Figure 12:**
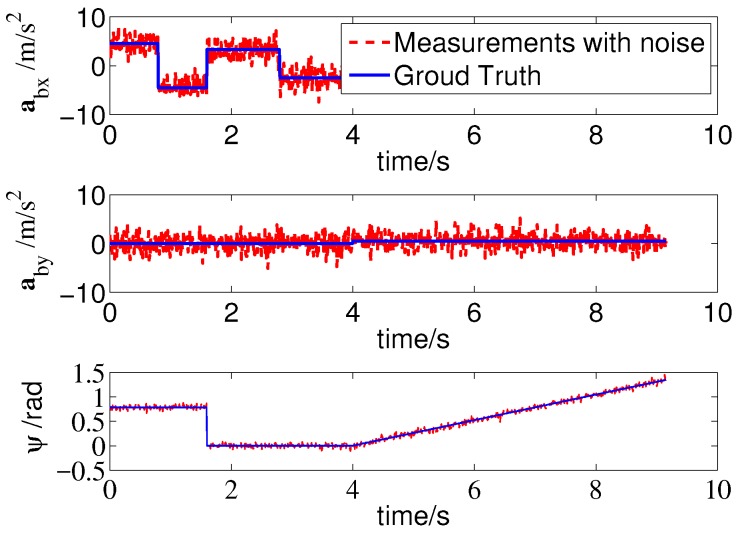
The true values and values with noise of inertial measurement unit (IMU) sensors.

**Figure 13:**
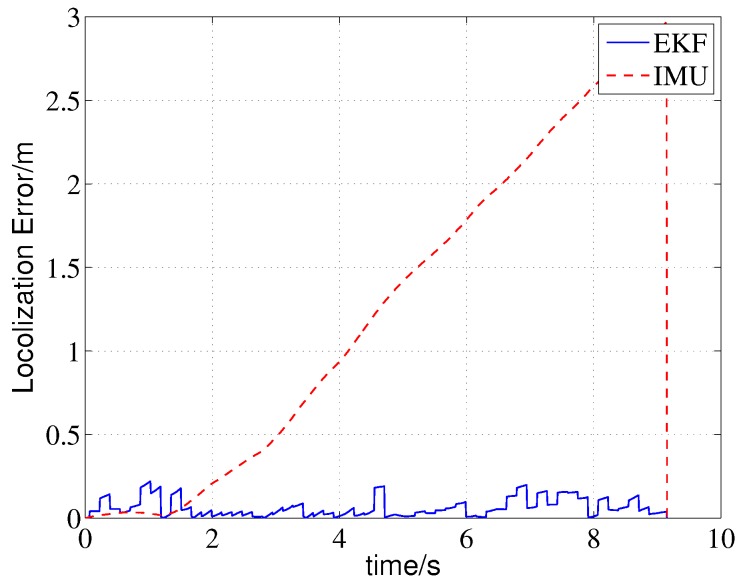
Localization errors.

**Figure 14:**
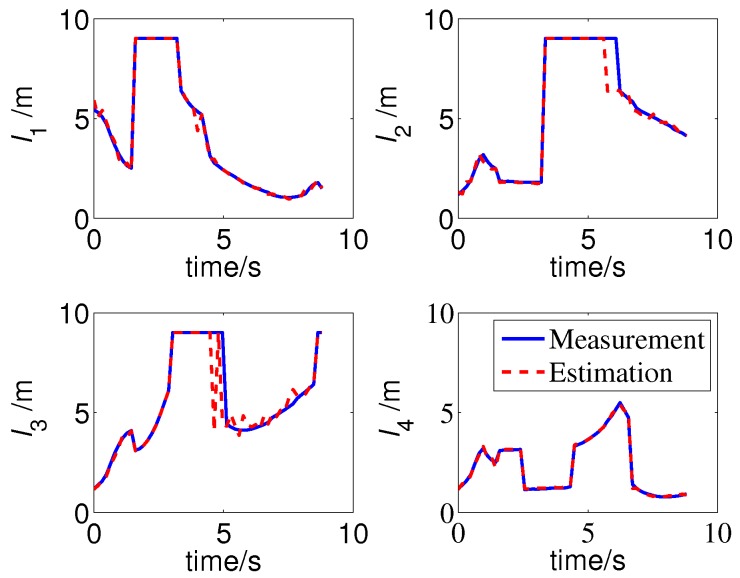
Measurements and multi-ray model estimations of ultrasonic sensors (simulation).

**Figure 15:**
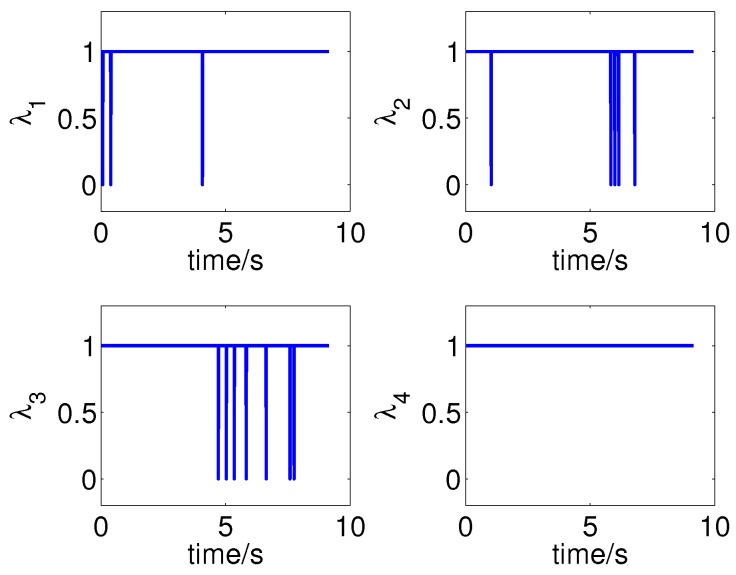
Parameters of jump filters.

The other parameters used in the simulation are presented in Table 5. The simulation results are shown in Figure 11, Figure 12, Figure 13, Figure 14 and Figure 15.

The actual trajectory of the MAV is shown by the solid line in Figure 11. The MAV first flew straight to the northeast and then straight north, and finally executed a turning maneuver. The true values of the IMU shown in Figure 12 illustrate that the MAV experienced many acceleration and deceleration events during the flight, and its heading angle also changed significantly with time.

The localization results based on the integral of IMU sensors and based on the proposed EKF approach are shown in Figure 11. The IMU position error increases over time due to the drift of the accelerometer, and the localization accuracy is poor. In contrast, the estimated locations of the EKF approach are very close to the actual trajectory. A quantitative error comparison is presented in Figure 13. The localization error of the proposed method is less than 0.25 m, while the IMU localization error increases cumulatively and finally approaches 2.8 m.

The measurements and multi-ray model estimations of the four sonar sensors are presented in Figure 14. The ultrasonic measurements have undergone multiple mutations over time; meanwhile, the mutation of the model estimations were not synchronized with the measurements due to localization errors, some differences even reached four meters, such as $l_{3}$ in 4.64 s. The activation of the jump filter is shown in Figure 15. In this case, errors of more than 0.3 m will be filtered out, and the threshold is selected based on the maximum possible cumulative error of the IMU during one sampling period of the sonar sensor. As shown in Figure 13, the difference between the estimations and measurements does not significantly affect the localization because of the correction of the jump filter.

**Figure 16:**
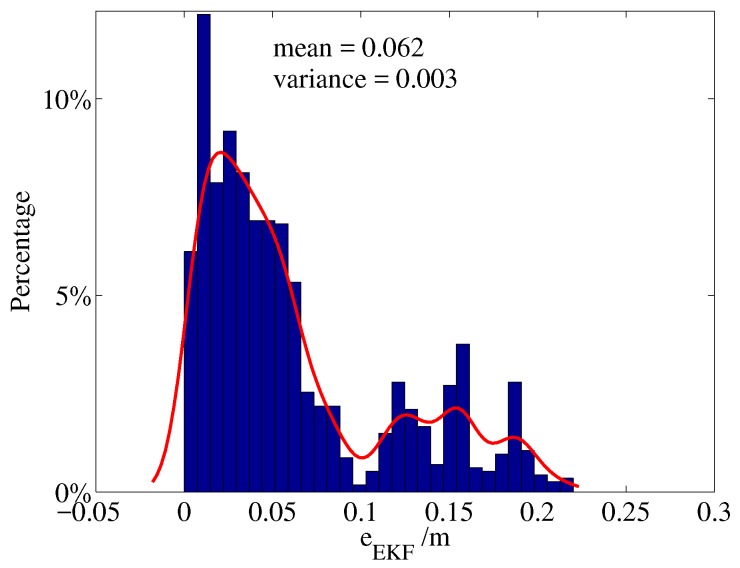
Distribution of EKF localization errors (Euclidean norm).

**Figure 17:**
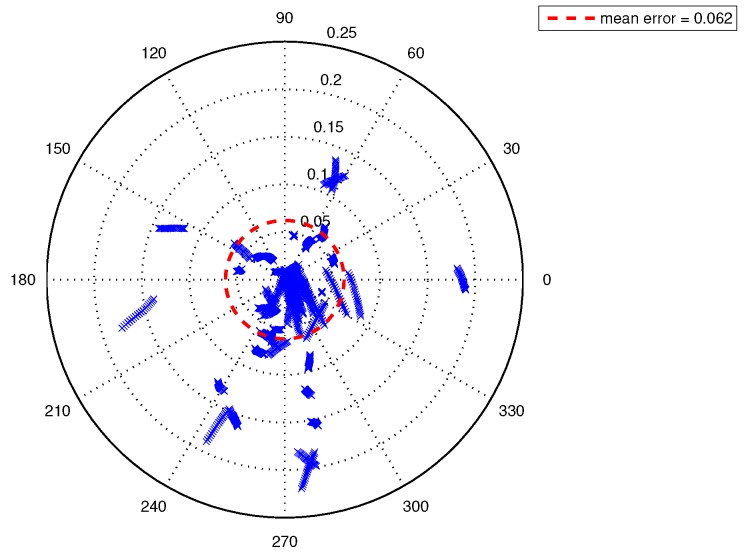
Distribution of EKF localization error vectors.

The statistical analysis of the localization error of EKF approach is shown in Figure 16 and Figure 17. Figure 16 shows the distribution of the Euclidean norm of EKF localization errors. The mean EKF localization error was 0.062 m and its variance was 0.003 $m^{2}$. The red line denotes a smoothing function fit of the error. The main components of the data are concentrated between 0 and 0.1 m, which is very close to a Rayleigh distribution. A small amount of data was distributed between 0.1 and 0.22 m, and this is due to the cumulative error caused by the asynchronous between the measurements and estimations. Figure 17 shows the distribution of the localization error vector, most of the data were less then the mean error, while a few data were close to 0.25 m.

### 6.2. Experimental Results

The proposed algorithm was implemented as an application of PX4 autopilot software. It acquired data from the IMU sensors every 8 mm and from four sonar measurements every 160 mm, and it reported the position of the MAV to the other applications. The ThalesII MAV platform was running the upgraded PX4 autopilot software.

**Figure 18:**
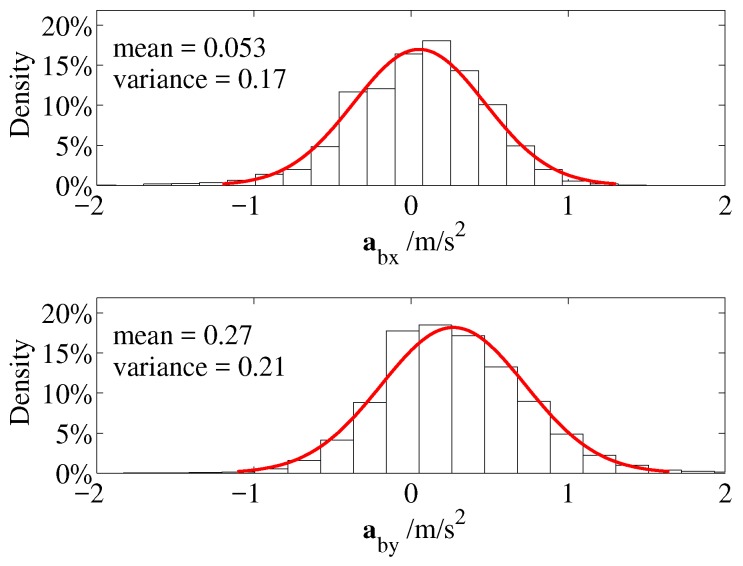
Distributions of IMU data.

In Figure 18 the red Gaussian describes the distributions of the acceleration values along $x_{b}$ and $y_{b}$ axes of the ThalesII MAV. The bias mean errors on $x_{b}$ and $y_{b}$ axes were 0.053 m/s${}^{2}$ and 0.27 m/s${}^{2}$, respectively, and the variations were 0.17 (m/s${}^{2}$)${}^{2}$ and 0.21 (m/s${}^{2}$)${}^{2}$, respectively. That shows the IMU sensors were not very accurate and may lead significant cumulative errors over time.

**Figure 19:**
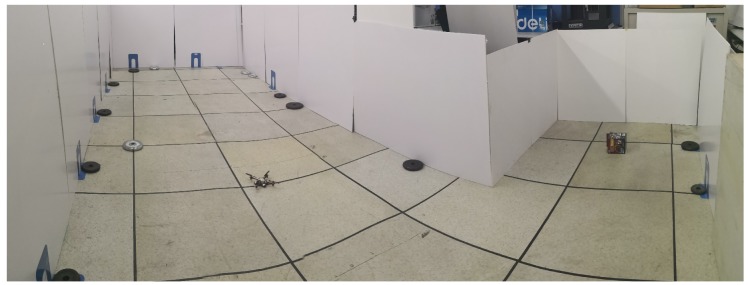
L-shaped test site.

An L-shaped experimental site was constructed using foam boards, as shown in Figure 19. Because we do not have a more accurate localization system, we used a preset path to validate the proposed approach. The test process is to first set a preset trajectory, then move the MAV as close as possible to the preset trajectory, and finally compare the positioning result with the preset trajectory. Note that the second step is achieved by manual operations; thus, it may lead to deviations between MAV’s actual position and the preset trajectory.

**Figure 20:**
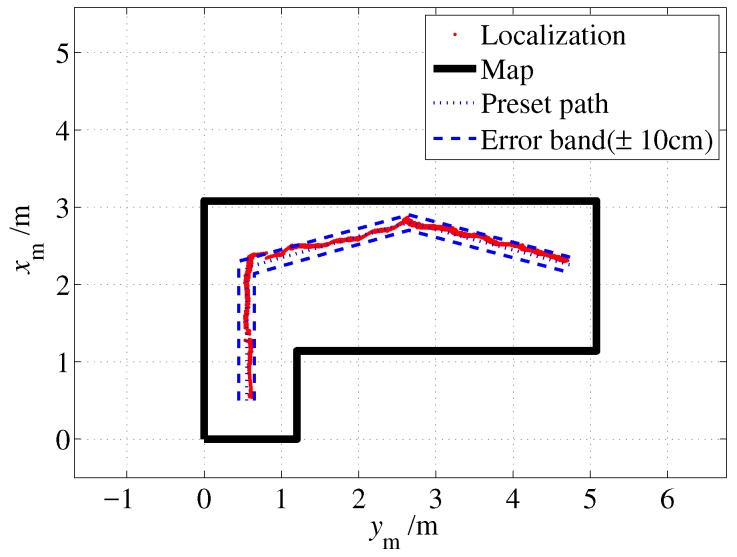
Localization result (without unmodeled obstacles).

As shown in Figure 20, the dotted line denotes the preset path, and it starts from the point (0.5, 0.55) and passes through two turns to reach the right end point (2.25, 4.75). A ±10 cm error band is also given by two dash lines, which is formed by two lines that are parallel to the preset path and each line is 10 cm away from the preset path. As shown in the figure, most of the localization outputs were within the error band which indicates that localization error does not exceed 20 cm. Considering the accuracy of human execution, the proposed approach can solve the indoor localization problem well.

**Figure 21:**
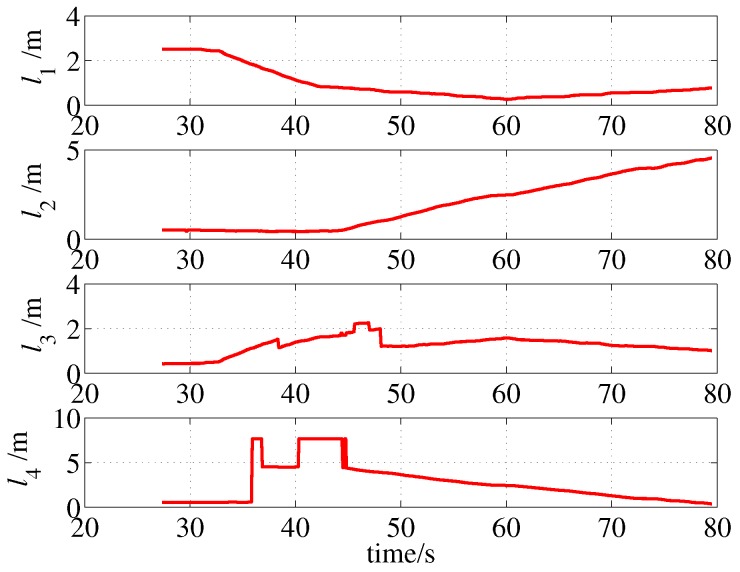
Sonar measurements (without unmodeled obstacles).

Figure 21 presents the measurements of the four MB1222 ultrasonic sensors. Note that the measurement data are stored as the localization application starts to run; thus, the recording time does not start from 0. As shown in Figure 21, the measurement may contain several jumps in the values when the ultrasonic reflected beam changes from one wall to the other. For example, the measurement of sonar no. 4, which points to the right side, jumped from 0.57 m to 7.65 m at approximately 36 s; this indicates that the MAV had just passed the first corner.

**Figure 22:**
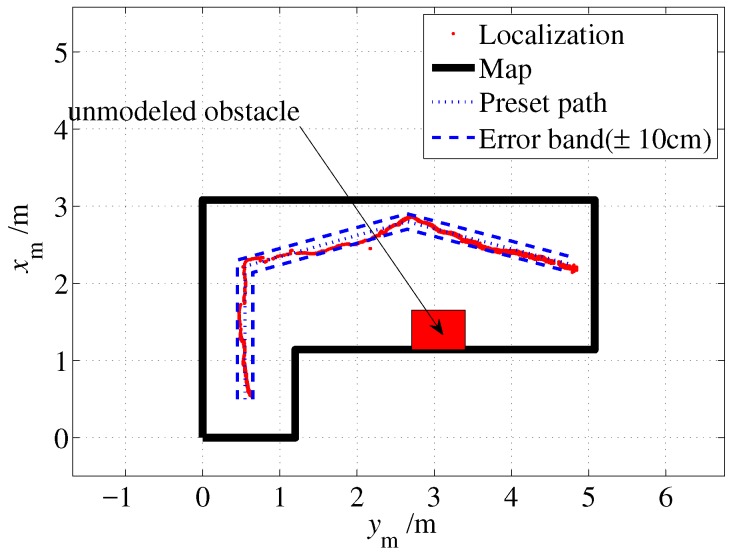
Localization result (with an unmodeled obstacle).

**Figure 23:**
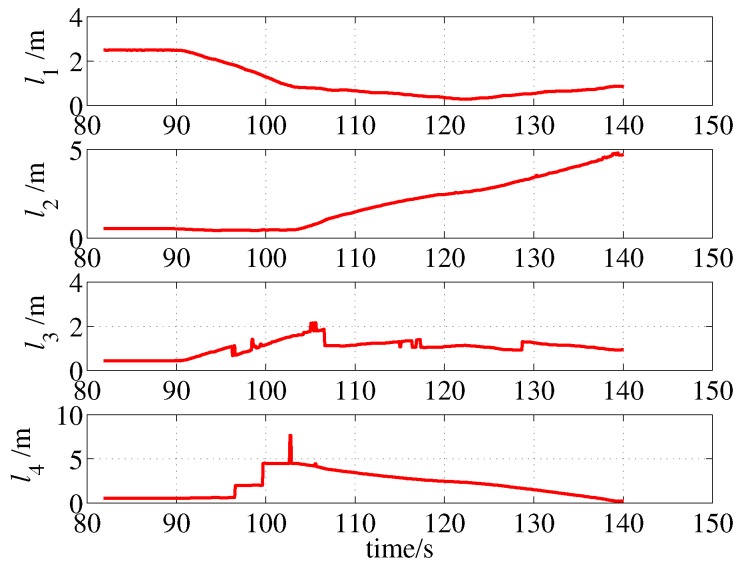
Sonar measurements (with an unmodeled obstacle).

In practice, the items in a room may change, which may adversely affect the localization algorithm. To test the adaptability of the algorithm to this situation, an unmodeled obstacle was placed in the test site. The obstacle was a box that was approximately 0.7 m long and 0.5 m wide. The test results are shown in Figure 22 and Figure 23. The proposed algorithm worked well with the unmodeled obstacle, as the localization results have not been significantly affected and stay within the error band.

## 7. Conclusions

In this paper, a novel beaconless indoor localization approach that relies on onboard ultrasonic sensors and IMU sensors is presented.

A multi-ray model for ultrasonic sensors is proposed. It approximates a beam pattern accurately while maintaining a low computational complexity, which makes it suitable to be applied to a light MAV. Then, a multi-ray modeling process has been provided based on the beam pattern of the MaxSonar MB1222 ultrasonic sensor. The comparative test validates that the proposed model has good fitness with the actual sensor for indoor localization.

Based on the multi-ray model, an EKF-based indoor localization method has been presented. The measurements of sonar sensors and IMU sensors are fuzed to achieve higher precision positioning. The jump filter is introduced to suppress the abnormal and significant difference between the estimations and measurements.

Simulations are presented to validate the proposed methods, and the results show it has a localization accuracy of approximately 20 cm. Afterwards, the proposed approach are applied to the ThalesII MAV, which is a small size and light weight platform. The results illustrate that its computational complexity is simple enough to run on the stm32 platform and positioning accuracy is also higher than 20 cm. An experimental test with an unmodeled obstacle shows the good robustness of proposed method, the localization results have not been significantly affected and stays within the error band.

Future work is to improve the algorithm for more complex indoor environments such as offices with many electric and electronic equipments, that may lead a large interference to the measurement of the magnetic compass.

## Figures and Tables

**Table 1:** Features of Thales II micro air vehicle (MAV) platform.

Total Weight	75 g
Wheelbase	13.5 cm
Battery	400 mA/3.7 V
Propulsion	820 Hollow cup motor/55 mm blade propeller
CPU	STM32F427
IMU Sensors	MPU6000/LSM303D/L3GD20H (8 ms sampling period)
Range Finder	MB1222 EZ2 (160 ms sampling period)
Transmission	Onboard ESP8285 WiFi module

**Table 2:** Measurements of MB1222 at various angles and distances.

$l_{t}$/cm	ψ/deg	0	5	10	15	20	25	30	35	40
*r*/cm										
30		27	27	27	26	26	25	24	24	–
60		57	57	56	55	54	53	50	49	–
90		86	86	85	84	82	80	78	78	–
120		116	115	114	113	110	109	106	106	–
150		146	145	144	142	140	139	136	–	–
250		247	245	244	245	243	–	–	–	–
350		346	345	344	–	–	–	–	–	–
450		447	446	444	–	–	–	–	–	–
550		547	545	543	–	–	–	–	–	–
590		587	–	–	–	–	–	–	–	–

mark “-” means that the sensor returned its maximum result, i.e., the reflection intensity did not reach the threshold of the sensor.

**Table 3:** Outputs of multi-ray model at various angles and distances.

$l_{m}-3$/cm	ψ/deg	0	5	10	15	20	25	30	35	40
*r*/cm										
30		27	27	27	26	25	25	24	24	23 *
60		57	57	56	55	54	53	52	50	49 *
90		87	87	86	84	82	81	79	77	75 *
120		117	117	115	113	111	109	106	104	101 *
150		147	146	145	143	142	140	138	136 *	134 *
250		247	246	245	243 *	242	240 *	238 *	236 *	234 *
350		347	346	344	342 *	339 *	337 *	335 *	332 *	329 *
450		447	445	443	440 *	437 *	434 *	431 *	428 *	424 *
550		547	546	544	542 *	540 *	538 *	536 *	534 *	531 *
590		587	587 *	587 *	587 *	587 *	587 *	587 *	587 *	587 *

* The corresponding measurement of the ultrasonic sensor is its maximum detection range.

**Table 4:** Errors of multi-ray model at various angles and distances.

$l_{e}$/cm	ψ/deg	0	5	10	15	20	25	30	35	40
*r*/cm										
30		0	0	0	0	1	0	0	0	–
60		0	0	0	0	0	0	−2	−1	–
90		−1	−1	−1	0	0	−1	−1	1	–
120		−1	−2	−1	0	−1	0	0	2	–
150		−1	−1	−1	−1	−2	−1	−2	–	–
250		0	−1	−1	2	1	–	–	–	–
350		−1	−1	0	–	–	–	–	–	–
450		0	1	1	–	–	–	–	–	–
550		0	−1	−1	–	–	–	–	–	–
590		0	–	–	–	–	–	–	–	–

**Table 5:** Simulation Parameters

Parameter	Value	Unit
$t_{\mathrm{imu}}$	8	ms
$t_{\mathrm{sonar}}$	160	ms
$V_{a}$	$2.2I_{2}$	$m/s^{2}$
$V_{l}$	$0.007^{2}I_{4}$	m
$V_{\psi }$	0.087	rad
ϵ	0.3	m
Q	diag([1,0.2,1,0.2])	
R	$0.007^{2}I_{4}$	
